# The Extraction of Teeth under Nitrous Oxide Anesthesia

**Published:** 1909-10-15

**Authors:** Alfred W. Hall


### THE EXTRACTION OF TEETH UNDER NITROUS OXIDE ANESTHESIA.

BY ALFRED W. HALL, D.D.S.

The relation between the extracting specialist and his colleagues of the dental profession should be that of perfect harmony, that the patients may receive the benefit of their combined efforts. The extractor is dependent on the general practitioner, and if he expects to receive courtesies it is no more than right that he should return them. It is an easy matter to let drop a word that will strengthen the dentist in the estimation of his patients, and it is the extractor's duty to do this unobtrusively.

The marvelous strides made in the past decade have so broadened the scope of dentistry that to be a master in every phase of the work would be an impossibility. Furthermore, the incompatibility of certain duties of the prrofession, such as the delicate technique required to insert a gold filling, and the nerve-racking business of extracting, is a strong incentive for the dentist to conserve his energies for more remunerative operations, and to leave the blood money for the specialist.

Frequently procrastination on the part of a prospective bridge patient, owing to the fear of suffering extractions, is the thief of the dentist's exchequer. This fear, however, can easily be alloyed by explaining that no pain is experienced when nitrous oxide is used, and that the anesthesia maintains only three or four minutes, leaving no after-effects.

Nitrous oxide is the safest general anesthetic known, as shown by the mortality statistics, and when it has been administered in connection with oxygen no deaths have occurred. I would much rather give nitrous oxide than to use cocaine, it being less treacherous.

There have been several inhalers devised for the administration of nitrous oxide. Some for administering through the nasal cavity only, some for administering through the oral cavity only, and some for administering through both the nasal and oral cavities at the same time. I can see little advantage in using the mouth inhaler, except, perhaps, in cases of men with full beards, as it is apt to interfere with the mouth prop and become troublesome.

For starting the anesthetic and carrying it to the surgical stage the facial inhaler is the most satisfactory, the gas being supplied at atmospheric pressure; but for prolonged anesthesia the nasal inhaler has every advantage, as it can be applied without obstructing the field of operation. In order that this inhaler should be effective the gas should be applied with pressure enough to force it back into the posterior nares; otherwise too much air will be taken in through the mouth, which of necessity must be held open.

Before starting anesthesia the nasal inhaler is placed upon the patient's forehead, where it is held by an elastic band passing around the head; the mouth prop is now put into position and the anesthetic started with the facial inhaler, the symptoms being carefully watched, which will appear as follows:

1. Drowsiness with cyanosis.

2. Inability to control voluntary muscles, as evidenced by the fact that the patient cannot open the eye when requested to do so, but the effort may be apparent.

3. Excitement in some nervous cases.

4. Loss of conjunctival reflex. This sympton cannot be relied upon, as often it will be absent, in which case it is well to raise the patient's arm, which will drop inert or show evidence of tonic spasm. These symptoms indicate that surgical anesthesia has ensued; however, the experienced anesthetist is more apt to be guided by the patient's breathing, than by any other one symptom. Stertorous breathing, caused by vibration of the ary*tæ*no-epiglottidean folds, is also an indication that surgical anesthesia has ensued.

The teeth may now be extracted, but if there are many of them, or if they are difficult, the operator should draw down the nasal inhaler into position and in this way the patient may be held under the anesthetic throughout a protracted operation, the assistant keeping careful watch and regulating the supply of nitrous oxide, occasionally allowing the patient to take one or more inhalations of air, thus intermitting the administration of gas and air as indicated by the symptoms, which should be anticipated.

The nitrous oxide and oxygen mixture is not indicated as prolonging the period of surgical anesthesia beyond that obtainable with nitrous oxide and air is useless to the extractor owing to the haemorrhagic engorgement of the air passages.

If the anesthetic is carried beyond the surgical stage, symptoms will occur as follows:

1. Loss of papillary reflex.
2. Fixation of eyeball.
3. Dilatation of pupils.
4. Temporary suspension of respiration.

If the latter continues long and oxygen is not used, the heart will be apt to show signs of weakness, but it does not often fail suddenly, as with chloroform, and gives time for oxygen artificial respiration. This may be accomplished by holding the hand over the mouth and forcing oxygen into the lungs through the nasal inhaler, care being taken not to use too much pressure. When the oxygen has expanded the lungs remove the hand from the mouth, also the inhaler from the nose, and press forcibly upon the chest, thus causing a forced expiration. Repeat this until patient shows signs of recovery.

As the respiration will nearly always show signs of embarrassment before the heart weakens, it is most necessary to watch it carefully, discontinuing the administration upon manifestations of stertor or other forms of pulmonary embarrassment.

Pregnant women take more kindly to chloroform than to nitrous oxide. Blumfield, Hewitt, and Buxton claim that nitrous oxide is contraindicated in pregnacy after the sixth month, and they advise against its administration even in the early stages of pregnacy if the subject is exceedingly excitable or prone to abortion. In these cases the history should always be inquired into, and the anesthetic should never be given so as to produce clonic spasms.

The general concensus of opinion is that both chloroform and ether are safer when given warm than when given cold; this also holds good with nitrous oxide. The reason in a l cases is identical, —when warm, the anesthetic is more readily absorbed and eliminated, consequently less danger of cumulative effects.

When extracting under nitrous oxide anesthesia it is necessary to do the work in a limited space of time without undue laceration of the soft tissue or injuring the maxillary bones. This can best be accomplished by examining the mouth before starting the anesthetic, and mapping out the work ahead. If the teeth are to be extracted on both jaws take the lower ones first, as it will often avoid the embarassing situation of having them obscured by blood.

Elevators are indispensable for work on the lower teeth. Their judicious use will in many cases lessen the chances of laceration. It would be impossible to clearly illustrate their use in detail without showing cuts, or the instruments themselves. To elevate an impacted, lower third molar it is necessary to insinuate the elevator under its mesial surface before it can be tipped out. A spear-shaped elevator is preferable for this work. For elevating the remaining root of a lower molar after one root has been extracted with the forceps, use a scoop-shaped elevator, placing it at the bottom of the socket from which the root has been extracted, and by a scooping motion break through the thin septum of process between the roots, thus engaging the remaining root and scooping it out. This method will lacerate less than forceps and is much quicker. These elevators come in rights and lefts, and are applicable on either side of the mouth, both for mesial and distal roots. For elevating lower roots whose rough edges have irritated the gum tissue, causing it to obscure them, use a push elevator, placing the blade under the edges of the soft tissue and causing it to follow along the buccal surface of the root, applying the force lingually, keeping the fingers of the left hand in position to protect the tongue in case undue force should be used.

Upper teeth as a rule are much easier to extract than the lowers, and for their extraction elevators are not indicated except in rare cases.

The number of teeth whose roots are in relation to the floor of the antrum are variable; the antrum may extend so far forward, especially in cases where the canine fossa is not pronounced, as to be in relation to all of the teeth of the true maxilla from the cuspid to the third molar. This fact should be borne in mind by the extractor, as in some cases the roots of these teeth extend fairly into the antrum, their ends being covered only by a membrane, hence the danger of pushing a root into the maxillary sinus.

When it is necessary to force through the process in order to extract a root, make a clean cut through over the root. This may be done with a narrow-beaked forcep with sharp edges. Place the beaks far enough up to make sure of the root, and then press them firmly together, using a very slight rotary motion to aid in cutting the bone, so that the root may be fairly engaged before attempting any lateral motion or pulling, otherwise a large piece of process is likely to be broken away and the floor of the antrum injured.

Do not attempt to force the beaks of a forcep between the alveolar process and the root; it is not likely to be suc cessful, and the root may be forced ahead of your forcep into the antrum.

Nature will cause the absorption of the process after extraction, and no harm will be done by carefully cutting through it in order to extract a badly broken down root. It is not to be inferred that I advocate cutting through the gums,—the forceps should be forced up under the gum tissue. This can be done with impunity, as the soft tissue will fall back to place and heal by first intention.—*The Dental Bur.*

